# Carbon nanotube counter electrode for high-efficient fibrous dye-sensitized solar cells

**DOI:** 10.1186/1556-276X-7-222

**Published:** 2012-04-17

**Authors:** Shuqing Huang, Huicheng Sun, Xiaoming Huang, Quanxin Zhang, Dongmei Li, Yanhong Luo, Qingbo Meng

**Affiliations:** 1 Key Laboratory for Renewable Energy, Key Laboratory for New Energy Materials and Devices, Institute of Physics (Beijing National Laboratory for Condensed Matter Physics), Chinese Academy of Sciences, Beijing, 100190, China

**Keywords:** Fibrous dye sensitized solar cells, Counter electrode, Carbon nanotubes, TiO_2_ nanotube arrays

## Abstract

High-efficient fibrous dye-sensitized solar cell with carbon nanotube (CNT) thin films as counter electrodes has been reported. The CNT films were fabricated by coating CNT paste or spraying CNT suspension solution on Ti wires. A fluorine tin oxide-coated CNT underlayer was used to improve the adherence of the CNT layer on Ti substrate for sprayed samples. The charge transfer catalytic behavior of fibrous CNT/Ti counter electrodes to the iodide/triiodide redox pair was carefully studied by electrochemical impedance and current-voltage measurement. The catalytic activity can be enhanced by increasing the amount of CNT loading on substrate. Both the efficiencies of fibrous dye-sensitized solar cells using paste coated and sprayed CNT films as counter electrodes are comparative to that using Pt wires, indicating the feasibility of CNT/Ti wires as fibrous counter electrode for superseding Pt wires.

## Background

Flexible dye-sensitized solar cells are the subject of active research as a good power supply for portable and integrated equipment [1]. Particularly, fibrous dye-sensitized solar cells (F-DSCs) based on various fibrous substrates (i.e., metal wires, optical fiber, carbon fiber, etc.) have attracted increasing attentions due to their unique structures for omnidirectional light absorption and weavable characteristic [2-4]. The metal-based F-DSCs have the advantages of low bulk resistance, low cost and easy fabrication. Recently, considerable efforts have been focused on the cost effective fibrous photoanode. It can be constructed by covering the metal wire substrate with titanium dioxide particle films, nanotube arrays, or nanowires films *via* simple dip-coating, spray techniques [5], or electrochemical-eroding Ti wires [6,7]. However, less attention has been paid to the fibrous counter electrode (CE). Commonly, the F-DSCs were assembled by twisting Pt wire CE and photoande together, and the conversion efficiency (*Eff*) of 5.8% and 1.86% could be achieved using liquid electrolyte [8] and solid electrolyte [9], respectively . However, the Pt wire was an extremely expensive CE for the practical applications of F-DSCs, which gave rise to the strong request for new cheaper materials to substitute Pt.

Carbon materials are considered to be excellent substitutes of Pt for their good reduction ability to tri-iodine ions in electrolyte. Various kinds of carbon, such as activated carbon [10], carbon nanotubes (CNT) [11-14], graphite [15], hard carbon spherules [16] and carbon black [17] have been studied. Due to their advantages of high electrical conductivity, chemical stability and high surface area, these carbon materials have been proven to be competitive with Pt as CE used in flat DSCs [18].

In this study, multiwalled CNT is firstly chosen as the catalytical material of CE for F-DSCs. Fibrous CEs are fabricated by two different methods, brushing CNT paste or spraying CNT suspensions on Ti wire substrate. Because the CE should be convolved on photoanode, the adherence of the active layer on wire substrate is of great importance. Fluorine tin oxide (FTO) was sprayed on CNT layer to improve the contact between CNTs and enhance the adherence of CNT layer which cycles to the surface of Ti wire. The impact of CNT loading amount and fabrication methods on the electrochemical catalytic activity of the CEs have been studied by electrochemical impedance spectra (EIS) and current-voltage measurement. The F-DSCs were assembled using dye-sensitized TiO_2_ nanotube (TNT)-coated Ti wire as photoanode and CNT-coated Ti wire as CE. The best energy conversion efficiency of 4.18% has been achieved under AM 1.5-G illumination.

## Methods

### Preparation of photoanode

The TiO_2_-nanotube photoanode was fabricated by anodic oxidation [8]. Ti wires (*Φ* = 0.3 mm, purity 99.7%) with length of 4 cm were first ultrasonically cleaned in ethanol and then electrochemically polished. The polished Ti wires were electrochemically eroded in ethylene glycol electrolyte containing 0.2 wt% NH_4_F and a small amount of deionized water, with an applied voltage of 50 V. The length of TNT can be adjusted by controlling the reaction time. The TNT-coated Ti wires were ultrasonically treated after anodic oxidation for several minutes and annealed at 450°C for 3 h for crystallization, then treated in 0.1 M TiCl_4_ aqueous solution at 70°C for 1 h, followed by re-annealing at 450°C for 30 min. After cooling down to 80°C, the TNT photoanodes were immediately immersed into 0.3 mM cis-bis (isothiocyanato) bis (2,2 = −bipyridyl-4,4 = −dicarboxylato) ruthenium(II) bistetrabutyl ammonium (N719, Dyesol, New South Wales, Australia) in ethanol for 24 h at room temperature.

### Fabrication of CNT CE

To prepare a viscous CNT paste, 0.5 g CNT powder (donated by Prof. Fei Wei from Tsinghua University, P.R. China [19]) was ultrasonically dispersed in 300 mL ethanol, then 10 ml of terpineol, 0.3 ml of ethyl cellulose alcoholic solution and 0.2 ml of titanium isopropoxide used as binder were added into the solution. To improve the dispersion of CNTs, the mixed paste was ball-milled for 24 h. The as-prepared CNT paste was brush coated on Ti wires (*Φ* = 0.1 mm). After drying at 80°C in the air for 30 min, CNT CEs fabricated by brush coating method (BCNT) were obtained by annealing at 385°C for 20 min with a heating rate of 2°C/min.

**Figure 1:**
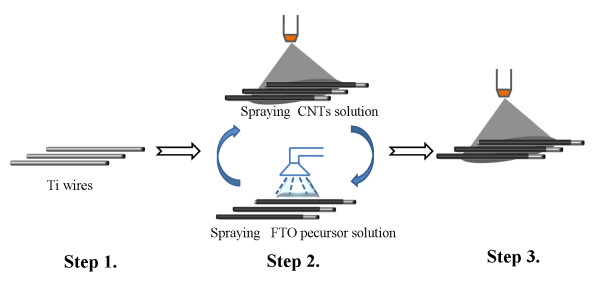
**The process scheme for preparing CNT fibrous CE by spraying method.** Step 1, placing Ti wires on substrate and raising the temperature of substrate to 400°C; step 2, spraying CNT solution for 40 s first, and then spraying FTO precursor on the samples for 3 min. This step was repeated three times, and the temperature was kept at 400°C; step 3, the temperature was cooled down to 300°C, and CNT suspension is continued to be sprayed.

To prepare CNT CEs by spraying method (SCNT), 0.5 g CNT powder was firstly treated with mixture solution of concentrated HNO_3_ and H_2_SO_4_ (*v/v* = 1/3) under 100°C for 1 h to obtain separated CNTs [20]. After washing with deionized water, the CNT suspension was filtrated, and the CNT powder was collected and dried. Then, purified CNT powder (10 mg) was ultrasonically dispersed into 100 mL deionized water for 2 h. For a good adherence to substrate, a FTO-coated CNT underlayer was introduced between CNT film and Ti substrate. The temperature of Ti wires substrate was kept at 400°C in the beginning of spraying to ensure the good crystallization of FTO on the surface of CNTs [21]. The as-prepared suspension was then sprayed on Ti wires (*Φ* = 0.1 mm) with a portable spray gun. After spraying CNT solution for 40 s, the ethanol precursor solution of FTO [21] was ultrasonically sprayed on the samples for 3 min, as shown in Figure 1. Spraying of CNT solution and FTO precursor solution was alternatively carried out for three circles. Then, the temperature was cooled down to 300°C, and CNT suspension was continued to be sprayed at different times.

### Cell assembly

Home-made reel equipment, which is able to automatically convolve the fibrous CE onto the photoanode, was used to assemble the F-DSCs. All the thread pitch distances of screwed CE were kept at 1 mm, according to our previous work [8]. To fix the fibrous solar cell and to avoid electrolyte drying out, two pieces of PET were used to clamp the samples. A small amount of electrolyte was dropped into the gap between the two pieces of PET, and the electrolyte would flow along with Ti wire due to the capillary force. The electrolyte was composed of 0.6 M methylhexylimidazolium iodide, 0.05 M of iodine, 0.5 M tert-butylpyridine, and 0.1 M of lithium iodide in 3-methoxypropionitrile.

### Measurements

The morphology of CE was investigated by scanning electron microscopy (SEM, FEI XL30 SFEG). Dummy cells were composed of two identical fibrous CEs laid parallel to each other with the distance of 0.5 mm and clamped by two pieces of glass. Electrolyte was injected into the space between the two CEs. EIS and current-voltage characteristic of dummy cells were applied to test the catalytic properties of CEs. For photovoltaic measurement, the F-DSCs were exposed to the illumination of standard simulated sunlight of 100 mW cm^−2^ (AM 1.5 G, Oriel 91160A, Newport Corporation, Beijing, China). A 20 mm × 1 mm mask was used to screen stray light and ensure the light to vertically illuminate onto solar cells. The photocurrent-voltage curves of F-DSCs were recorded by IM6e electrochemical work station (Zahner Co., Germany). The frequency of the superimposed signal is from 100 kHz to 0.1 Hz with an AC amplitude of 10 mV. The effective illuminated area of one fibrous solar cell was calculated as the product of the Ti wire diameter and the cell length (2 cm), which was 0.06 cm^2^ for Ti wire with diameter of 0.3 mm.

## Results and discussions

**Figure 2:**
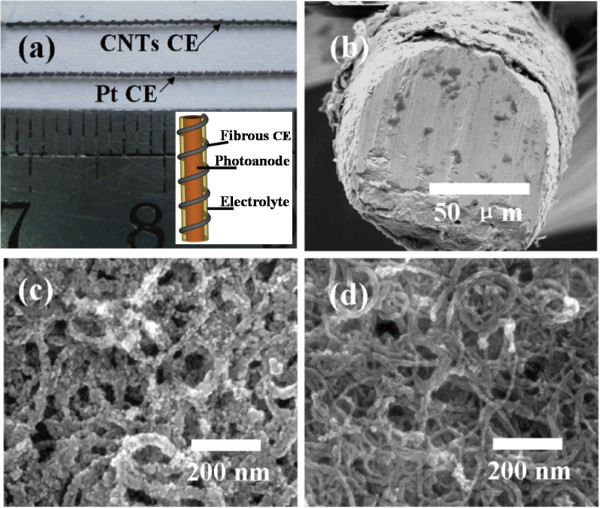
**Photo of F-DSCs and SEM images of CNT CEs.** (**a**) The photo of two F-DSCs using CNTs and Pt as CEs, respectively. Inset is a schematic structure of F-DSCs. (**b**) The side view SEM image of a BCNT fibrous CE; (**c**) and (**d**) are top view SEM images of FTO-coated CNTs and bare CNTs, respectively.

Figure 2 shows the photo of two F-DSCs using Pt wire and CNTs/Ti wire as CEs, respectively, and SEM images of CNT CEs. As seen from Figure 2a, both Pt and CNTs/Ti CEs were convolved around the sensitized TNT anode with uniform screw pitch which ensures the good reproducibility of the experiments. For clarity, a schematic image of F-DSCs is given in the inset of Figure 2a. Figure 2b shows that one thin layer of CNTs with a thickness of 4 μm is coated on Ti wire by brush coating method. However, the SCNTs are much thinner than the BCNT, and the thickness of SCNT is very difficult to be detected from SEM measurement. FTO crystals are obviously covering the CNTs after spraying the FTO precursor (Figure 2c). The FTO-coated CNT layers act as adhesive to improve the contact between CNTs and substrate. Figure 2d shows the CNT network which provides a large surface area with CNT diameters ranging from 10 nm to 20nm.

The catalytic ability of carbon electrode is mainly influenced by the active surface area of the carbon layer. Increasing the loading amount of carbon materials on the substrate will directly raise the surface area, providing more sites for I_3_^−^ reduction and, subsequently, improve the performance of the CEs. Normally, the thicknesses of the carbon layer on CEs are varied from several microns to more than 100 μm [15]. However, too thick CNT layers would be easily crashed due to the distortion and extension during the convolving step in the assembly process of F-DSCs. Thus, the carbon layer thickness should be carefully controlled.

**Figure 3:**
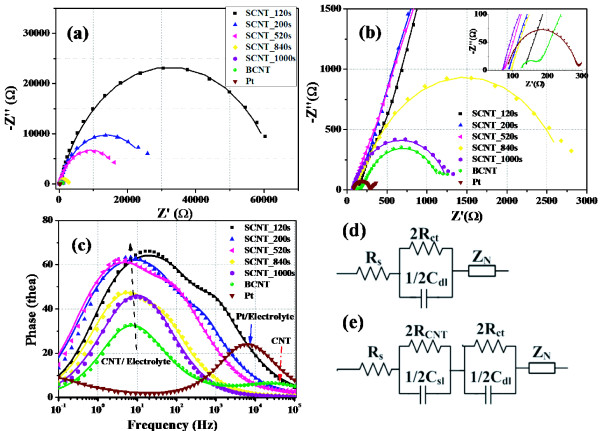
**EIS results.** (**a**) and (**b**) are the Nyquist plots of EIS of symmetrical dummy cells fabricated with different fibrous electrodes; (b) is the amplificatory figure of (a); the inset of (b) shows the detail of overlapped part in high frequency region of Nyquist plots; (**c**) shows the Bode-phase plots. (**d**) is the equivalent circuits of symmetrical dummy cells except for BCNT-BCNT type and (**e**) is the equivalent circuit of the BCNT-BCNT dummy cell, where *R*_s_ is the serial resistance, *R*_ct_ is the electron transfer resistance at the interface of electrode/electrolyte, *C*_dl_ is the double layer capacitance of the electrode/electrolyte interface, *Z*_N_ is the Nernst diffusion impedance of electrolyte, *R*_CNT_ is the electron transfer resistance in solid CNT net and *C*_sl_ is the capacitance of CNT net itself. Dots are the experimental data and solid lines are the fitted results.

To investigate the influence of the CNT loading amount on the catalytic activity of SCNTs, EIS measurements of dummy cells assembled by two identical electrodes have been carried out. Figure 3a,b shows the Nyquist plots of EIS results, and Bode-phase plots are illustrated in Figure 3c. For a Pt-Pt symmetrical cell, one semicircle and a beeline appeared as the results of the Pt/electrolyte interfacial charge transfer at around 1–100 kHz and the semi-infinite diffusion of electrolyte at low frequency domain (<1 Hz), respectively. All the Nyquist plots of CNT-CNT dummy cells show inherent semicircles near the frequency region of 1–100 Hz, representing the charge transfer at the CNTs/electrolyte interface [14]. However, the electrolyte diffusion impedances (*Z*_N_) cannot be easily distinguished in this case because of the overlap with the middle-frequency peaks. The characteristic peak frequency in Figure 3c is related to the charge transfer rate. The peak frequency of the charge transfer at CNT/electrolyte interface is smaller than that at the Pt/electrolyte interface, suggesting that the charge-transfer process at the CNT/electrolyte interfaces is slower than that at the Pt/electrolyte interface. A unique impedance of BCNT/BCNT dummy cell with a very fast time constant around 50 kHz was observed in Figure 3b, which is most possibly the result of charge transfer within solid CNTs [14]. The EIS results can be fitted by the equivalent circuit exhibited in Figure 3d,e and the fitted parameters are summarized in Table 1.

**Table 1:** **The fitted parameters of EIS results shown in Figure**3

**Sample**	**SCNT_120s**	**SCNT_200s**	**SCNT_520s**	**SCNT_840s**	**SCNT_1,000s**	**BCNT**	**Pt**
*R*_ct_ (Ω ·cm^2^)	1,257	542	355	52	22	16	3.75
*C*_dl_ (μF)	4.34	20.1	33.5	100	148	139	2.11

SCNT, CNT CEs prepared by spraying method; BCNT, CNT CEs fabricated by brush coating method; *R*_ct_, electron transfer resistance at the interface of electrode/electrolyte; *C*_dl_, double layer capacitance of the electrode/electrolyte interface.

The electron transfer resistance (*R*_ct_) at the interface of CNTs/electrolyte reduced with the spray time increased, and the *R*_ct_ of BCNT is the lowest among the CNT CEs. For the FTO-coated CNT layer (SCNT_120s), it showed a very large *R*_ct_ of 1,257 Ω ·cm^2^ because of very poor catalytic performance of FTO. With increased spray time, the *R*_ct_ of CE reduced as the result of increased amount of CNT loaded on substrate. It could be noted that the capacitance value *C*_dl_ inversely increased, indicating an increased inner surface area of the SCNT CEs. Exchange current density (*J*_*0*_) can be calculated from *R*_ct_ according to the following equation, which shows the electron releasing ability of CE at the equilibrium state.

(1) $$J_{0}=\frac{RT}{nFR_{\text{ct}}}$$

SCNT_1,000s and BCNT show relatively low *R*_ct_ of 22 Ω ·cm^2^ and 16 Ω ·cm^2^, respectively, giving rise to high *J*_0_ values, two orders larger than *J*_0_ of SCNT_120s. Thus, they have much better catalytic activity. Accordingly, the Pt CE shows the lowest *R*_ct_ of 3.75 Ω ·cm^2^ and best catalytic performance.

**Figure 4:**
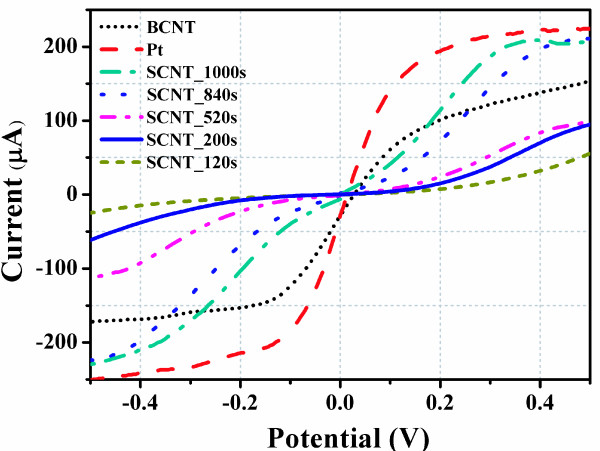
Current-voltage curves of the symmetrical dummy cells fabricated with different CEs.

To further demonstrate the catalytic activity of various CEs, current-voltage characteristics of the symmetrical dummy cells are investigated, and the results are shown in Figure 4. Pt shows excellent catalytic activity for the I_3_^−^ reduction with a sharp slope of current-voltage curve at small bias region. Comparing with Pt electrode, the current is lower for SCNT electrode; a distinct current flat between −0.2 V to 0.2 V was observed in the current-voltage curves of the SCNT samples with spraying time less than 840 s. The slope of current-voltage curves at low-voltage range becomes larger with the increasing spraying time because of the improved CNT loading. The increasing current of SCNT electrode indicates an improving catalytic activity, and this is in good accordance with the results of EIS.

**Figure 5:**
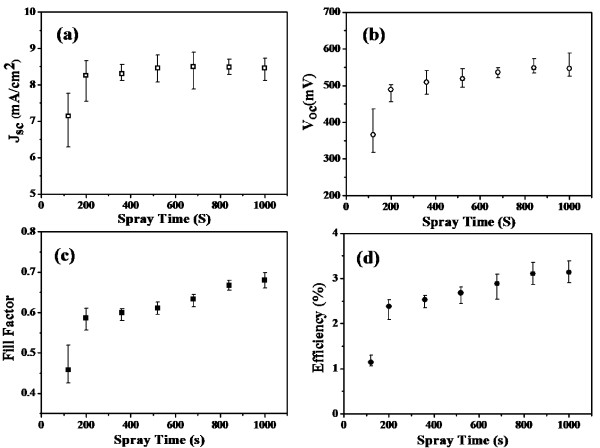
**The change rules of photovoltaic parameters of F-DSCs on the function of spraying time.** The F-DSCs use SCNT as CEs and the 18 μm TNT as photoanode. The parameters are (**a**) *J*_sc_ (short circuit current density), (**b**) *V*_oc_ (open circuit voltage), (**c**) *FF* (fill factor) and (**d**) *Eff* (conversion efficiency).

In order to investigate the influence of CNT loading amount on Ti wires on photovoltaic performance of F-DSCs, the change of each photovoltaic parameter of F-DSCs using SCNT as CE with different spraying time was systemically studied. Figure 5 shows the change rules and each average value was summarized *via* five F-DSCs samples with the same CE. As seen from Figure 5, the short circuit current density (*J*_sc_) increases very fast at early spraying stage and keeps almost constant after 360-s spraying. The open circuit voltage (*V*_oc_), fill factor (*FF*) and *Eff* also significantly increase at first and, then, gradually increase until 1,000 s. As discussed above, it is obvious that the improvement of *FF* from 0.46 to 0.68 is attributed to the decreased *R*_ct_ of the SCNT CEs. The increase of *FF* could also be explained by the different catalytic activity of SCNT CEs. The current-voltage curves of SCNT samples with lower CNT loading amount have smaller slopes according to Figure 4. To maintain the same photocurrent, higher overpotential is required for SCNT with shorter spraying time, which leads to more potential loss at CE side and result in lower *FF*[22].

In most cases, *J*_sc_ is independent with the *R*_ct_ of CE, except for the case of SCNT_120s with a FTO-sprayed surface. The *I*_sc_ can be represented by the following equation [23]:

(2) $$I_{\mathrm{sc}}=I_{\mathrm{ph}}-I_{0}\cdot [\exp(e\cdot I_{\mathrm{sc}}\cdot R_{s}/nkT)-1]-I_{\mathrm{sc}}\cdot R_{s}/R_{\mathrm{sh}}$$

Where *I*_ph_ is the short circuit current without parasitic resistance, *I*_0_ is the reverse saturated current, *R*_s_ is the series resistance and *R*_sh_ is the shunt resistance. *R*_ct_ at counter electrode introduces large *R*_s_ equivalently. Normally, *I*_0_ is much smaller than *I*_ph_ in several orders and *R*_sh_ is much larger than *R*_s_. So, the change of *R*_ct_ could not lead to predominant change of the exponential part and *R*_s_/*R*_sh_. However, if the *R*_ct_ is large enough, it will cause obvious decrease of photocurrent at short-circuit condition, which is the same as the cases of SCNT samples with only FTO coated on the CNT layer or very thin CNT layer.

The poor catalytic activity of FTO-coated CNT underlayer also leads to lower *V*_oc_[17], spraying CNTs on this underlayer make *V*_oc_ increased quickly and it changed very slowly after spraying time of 360 s. Finally, the best average value of *Eff* of 3.14% is obtained while the spraying time is 1,000 s.

**Figure 6:**
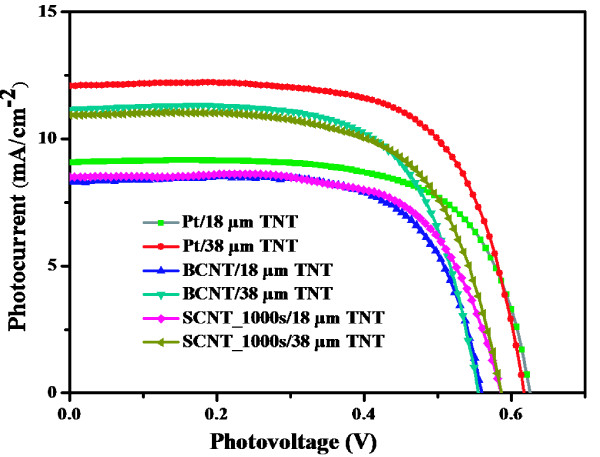
**Photocurrent-voltage curves of F-DSCs.** These F-DSCs use SCNT (spray time 1,000 s), BCNT and Pt as CEs with 18 μm TNT and 38 μm TNT as photoanode.

Figure 6 shows the effect of the nanotube length of the TNT photoanode on the photovoltaic performance of F-DSCs. The photovoltaic parameters of each curves was summarized in Table 2. The F-DSCs with BCNT and SCNT (spraying time 1,000 s) CEs have almost the same photovoltaic behavior, except for the *V*_oc_. For BCNT CEs, some CNT particles would drop from substrate and absorb onto the photoanode during assembly, bringing more recombination loss between photoanode and electrolyte, which is a possible reason for the lower *V*_oc_ of BCNT-based solar cells. All the F-DSCs with Pt wire as CEs have higher *J*_sc_ and *V*_oc_ than that of cells with CNT CE. The reflectance effect of the smooth surface of Pt wire is considered as one reason for the higher *J*_sc_ and *V*_oc_. According to our previous work, the percentage of area covered by CE of the whole photoanode is approximately 10% while the thread pitch of CE is 1 mm [8]. When the angle between the orientation of incident light and the normal of Pt wire surface is larger than 45°, the incident light can be reflect by Pt wire to the surface of photoanode, which is about 5% of the whole incident light. Consequently, the local light intensity for F-DSCs with Pt CE is higher than that for the ones with CNT CEs.

**Table 2:** **Photovoltaic parameters of photocurrent-voltage curves shown in Figure**6

**CE**	***J***_sc_**(mA cm**^−2^**)**	***V***_oc_**(*V*)**	***FF***	***Eff*(%)**	**Length of TNT (μm)**
BCNT	8.32	0.563	0.690	3.24	18
SCNT_1,000s	8.49	0.588	0.680	3.40	18
Pt	9.08	0.628	0.676	3.85	18
BCNT	11.12	0.556	0.670	4.14	38
SCNT_1,000s	10.95	0.588	0.649	4.18	38
Pt	12.06	0.620	0.676	5.05	38

BCNT, CNT CEs fabricated by brush coating method; SCNT, CNT CEs by spraying method; *J*_sc_, short circuit current density; *V*_oc_, open circuit voltage; *FF*, fill factor; *Eff*, conversion efficiency; TNT, TiO_2_ nanotube.

According to our previous work [8], the efficiency of F-DSC would increase along with the length of TNT when the length is shorter than 40 μm. Further increasing the length, the TNTs would easily collapse. Photoanode with 38 μm TNT was used to obtain a relatively high *Eff.* As seen from Table 2, the *Eff*s are 4.14% and 4.18% for F-DSCs using BCNT and SCNT as CEs, respectively, both reach 80% of the *Eff* of the cell with Pt CE. This result indicates the potential application of the CNT-coated Ti wires as the CE for F-DSCs. Furthermore, the SCNT CEs with FTO-coated CNT underlayer have better connection between CNT film and substrate than that of BCNT CEs. They can be reused because the CNTs would not easily crush from the substrate by distortion, while the SCNT samples with thicker activated layers show contrary result. Therefore, the CNT CEs fabricated by spray method are more practical.

## Conclusions

The catalytic characters of CNT fibrous CEs prepared by brush coating and spraying methods have been carefully investigated by electrochemical impedance technique and current-voltage measurement. The catalytic activity of CNT films is enhanced with CNT loading amount as the results of the increasing inner surface area of CNT films. However, too thick CNT film which is prepared by brush coating method would lead to CNT particles pilling from substrate during cell assembly process, indicating that the CNT CEs fabricated by spray method are more practical. Both the efficiencies of F-DSCs with CNT CEs prepared by brush coating and spraying methods (spraying time is 1,000 s) exceeded 4% and reached 80% of that of F-DSCs with Pt CE, indicating the application potential of CNT fibrous CE as the substitute of Pt wire.

## Competing interests

The authors declare that they have no competing interests.

## Authors’ contributions

The work presented here was carried out in collaboration of all authors. QM and SH defined the research theme. SH designed methods and experiments, carried out the laboratory experiments, analyzed the data, interpreted the results and wrote the paper. HS, XH, and QZ co-worked on associated data collection and their interpretation. YL and DL co-designed the experiments and discussed the analyses, interpretation and presentation. All authors have contributed to the revision of the manuscript and have read and approved the final manuscript.
